# Randomized controlled trials in ophthalmology: a bibliometric study

**DOI:** 10.12688/f1000research.20673.1

**Published:** 2019-10-04

**Authors:** Saif Aldeen AlRyalat, Areen Abukahel, Khaled Ali Elubous

**Affiliations:** 1 Department of Ophthalmology, University of Jordan, Amman, Jordan

**Keywords:** Ophthalmology; Randomized Controlled Trials; PubMed; Retina; Journals; Bibliometrics.

## Abstract

**Background:** Randomized controlled trials (RCTs) are situated at the top of hierarchy of evidence-based medicine, where its number and quality are important in the assessment of quality of evidence in a medical field. In this study, we aim to assess the status of RCTs in Ophthalmology.

**Methods:** On 15 ^th^ of May 2019, we performed a PubMed search for randomized controlled trials published in the field of ophthalmology using relevant filters and search terms. We categorized the results into specific topics in ophthalmology according to Medical Subject Heading (MeSH) database classification system. We used Altmetric explorer to identify journals and articles with the highest number of RCTs and highest citations.

**Results:** We found a total of 540,427 publications in the field of ophthalmology, of which only 11,634 (2.15%) of them were RCTs. ‘Retinal diseases’ was the topic with the highest number of RCTs, followed by ‘glaucoma’ and ‘conjunctival diseases’. The trial with highest number of citations was on retinal diseases. Only around 18% of all ophthalmology RCTs are published in the top 10 ophthalmology journals, with a maximum percentage of RCTs was (5.53%) published in *Ophthalmology*.

**Conclusion:** RCTs in ophthalmology primarily concern the retina, glaucoma, and a few other sub-topics, with little focus on sclera, orbit, and the eyelids. Most of the high impact RCTs are published in non-ophthalmology journals.

## Introduction

Since the conception of the term “evidence-based medicine” in clinical practice in 1992 ^1^, where well-conducted randomized controlled trials (RCTs) are situated at the top of hierarchy of evidence, there has been an emphasis on accepting high quality evidence in terms of RCTs in clinical practice. Moreover, previous reports showed that RCTs have generally higher methodological rigor than observational studies ^2^. However, despite the rapid growth in ophthalmology literature in the recent years, this growth has not been paralleled by a growth in the quality of evidence ^3^. This is evident by the number of Cochrane reviews that don’t include any RCTs (i.e. empty review), which were estimated to be half of the total reviews on Cochrane Eyes and Vision in 2013 ^4^. In this study, we aim to assess the status of RCTs in ophthalmology, and will focus on publishing trends for RCTs in ophthalmology in the recent years with regards to different ophthalmology topics.

## Methods

### PubMed search strategy

On 15 ^th^ of May 2019, we performed a PubMed search for randomized controlled trials published in the field of ophthalmology. We used the following search filters:

- Ophthalmology studies: eye diseases [MeSH Terms]
- RCT: Randomized Controlled Trial [Publication Type]

To categorize the results into specific topics in ophthalmology, we used the Medical Subject Heading (MeSH) database to identify the topics within ophthalmology, where the following were included:

- Orbital Diseases
- Conjunctival Diseases
- Corneal Diseases
- Eyelid Diseases
- Lacrimal Apparatus Diseases
- Lens Diseases
- Glaucoma
- Refractive Errors
- Scleral Diseases
- Uveal Diseases
- Retinal Diseases

For each topic, we added the query as a MeSH term to the search to identify relevant articles (e.g. Orbital diseases[Mesh Terms]. It is worth noting that trials might be categorized in more than one topic.

To identify journals with the highest number of RCTs and top articles with highest citations, we used Altmetric database, where we inputted the PubMed query we used in the PubMed search in the search field; the database yielded citation information about searched articles along with information about the journals these articles published previously ^5^.

### Variables

For each RCT, we extracted data regarding the topic of the study and categorized them into the following: RCTs per year, percentage of each sub-specialty, Articles per sub-specialty per year, Top 10 journals with their respective data, Top 10 articles with highest dimensions citations

## Results

### Ophthalmology RCTs

**Figure 1.**
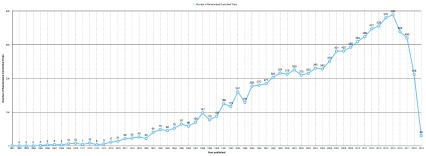
Number of randomized controlled trials published per year in the field of ophthalmology from 1961 to May 2019.

A total of 540,427 publications in the field of ophthalmology were identified, of which only 11,634 (2.15%) of them were RCTs. There was a total of 482,791 RCT identified in all disciplines, of which only 2.4% are in the field of ophthalmology. Of these trials, 124 were phase 1 trials, 270 were phase 2 trials, 380 were phase 3 trials, and 42 phase 4 trials; all others did not have phases. Number of RCTs peaked in 2015 with a total of 583 trials. Figure 1 shows the trend in number of RCTs in the field of ophthalmology.

### RCTs in each ophthalmology topic

**Figure 2.**
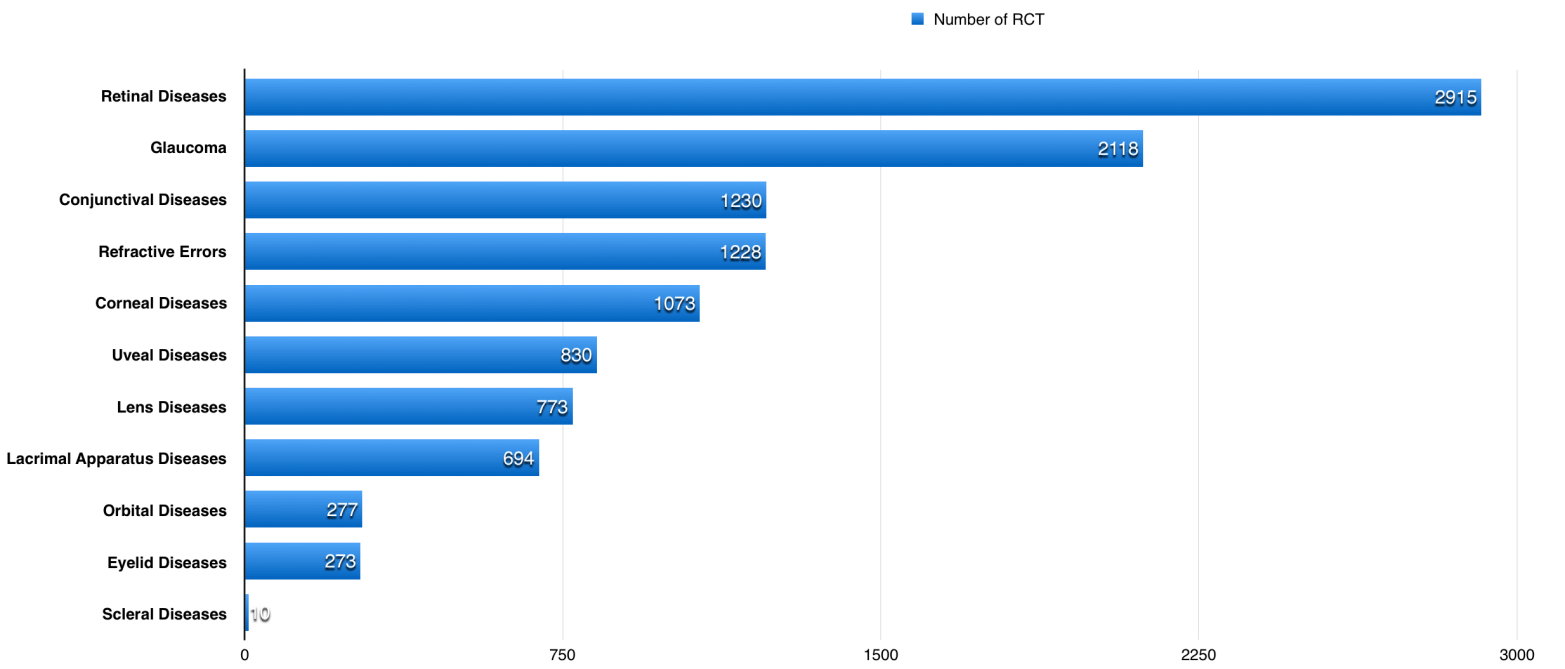
The number of randomized controlled trials (RCTs) for each topic in ophthalmology.

‘Retinal diseases’ is the topic with the highest number of RCTs, with a total of 2915 trials, followed by ‘glaucoma’, with 2118 trials, and ‘conjunctival diseases’, with 1230 trials. Figure 2 details the number of trials for each topic.

### Top RCTs and ophthalmology journals publishing RCTs

The trial with highest number of citations discussed retinal complications of diabetes mellitus entitled “The Effect of Intensive Treatment of Diabetes on the Development and Progression of Long-Term Complications in Insulin-Dependent Diabetes Mellitus”, published in *The New England Journal of Medicine* ^6^. Table 1 details the top 10 RCTs with highest citations. A total of 2090 (18%) of the RCTs were published in 10 journals, with “Ophthalmology” being the top journal with highest number of RCT published in it (643 RCTs). Table 2 details the top 10 journals with highest number of RCTs published in them.

**Table 1.** The 10 randomized controlled trials with the highest number of citations.

Number	Citations	Title	Journal	Publication Date	Reference	OA Status
1	16741	The Effect of Intensive Treatment of Diabetes on the Development and Progression of Long-Term Complications in Insulin-Dependent Diabetes Mellitus	New England Journal of Medicine	1993	6	FALSE
2	5177	Tight blood pressure control and risk of macrovascular and microvascular complications in type 2†diabetes: UKPDS 38 ^12^	British Medical Journal	1998	12	TRUE
3	3623	Ranibizumab for Neovascular Age-Related Macular Degeneration	New England Journal of Medicine	2006	13	FALSE
4	2343	Ranibizumab versus Verteporfin for Neovascular Age-Related Macular Degeneration	New England Journal of Medicine	2006	14	FALSE
5	2239	The Ocular Hypertension Treatment Study	Archives of Ophthalmology	2002	15	TRUE
6	1736	Pegaptanib for Neovascular Age-Related Macular Degeneration	New England Journal of Medicine	2004	16	TRUE
7	1683	The advanced glaucoma intervention study (AGIS): 7. the relationship between control of intraocular pressure and visual field deterioration	American Journal of Ophthalmology	2000	17	FALSE
8	1641	The Ocular Hypertension Treatment Study	Archives of Ophthalmology	2002	18	TRUE
9	1640	Whole-Body Hypothermia for Neonates with Hypoxic-Ischemic Encephalopathy	New England Journal of Medicine	2005	19	FALSE
10	1526	Grading Diabetic Retinopathy from Stereoscopic Color Fundus Photographs - An Extension of the Modified Airlie House Classification	Ophthalmology	1991	20	FALSE

OA, open access.

**Table 2.** The 10 ophthalmology journals with the highest number of randomized controlled trials (RCTs) and proportion of RCTs of total ophthalmology RCTs published in each.

Journal	Number of RCTs	Percentage from total ophthalmology RCT
***Ophthalmology***	643	5.53%
***American Journal of Ophthalmology***	333	2.86%
***British Journal of Ophthalmology***	246	2.11%
***Investigative Ophthalmology & Visual Science***	163	1.40%
***Archives of Ophthalmology***	157	1.35%
***Journal of Cataract & Refractive Surgery***	149	1.28%
***Retina***	106	0.91%
***JAMA Ophthalmology***	103	0.89%
***Optometry and Vision Science***	97	0.83%
***Journal of Glaucoma***	93	0.80%
Total	2090	17.96%

## Discussion

In the current study, we observed a peak in the annual number of RCTs on 2015, after which a steady decrease observed till 2018. Retinal diseases is the topic with the highest number of RCTs, followed by glaucoma and conjunctival diseases. The trial with highest citation was on retinal diseases and was published in *The New England Journal of Medicine*, where also other top cited trials were published in general non-ophthalmology journals. The total RCTs published in top 100 ophthalmology journals was only 2090 (17.96%).

In general, there has been an increase in the number of RCTs in ophthalmology since the late 1990s. In a study assessing the frequency of prospective studies published in the *American Journal of Ophthalmology* and *British Journal of Ophthalmology*, they found an increase from 1% to 12% during the years 1980 to 1999 ^7^. We observed a low number of RCTs among the ophthalmology literature, a percentage that didn’t exceed 2.5% of the overall ophthalmology literature. In a previous study assessing the frequency of RCTs published in the major four ophthalmology journals, they found that only around 3.5% of their annual publications are RCTS ^8^. Moreover, we found that only around 18% of all ophthalmology RCTs are published in the top 10 ophthalmology journals, with the most RCTs (5.53%) published in *Ophthalmology*. In a study that reviewed risk of bias in RCTs published in major ophthalmology journals found that a risk of bias was observed in 29.4% of published RCTs ^9^. In another study that assessed fragility of RCT’s that included the comparison between two groups found a high proportion of fragile results in ophthalmology RCTs ^10^. In a study that assessed types of articles published in core pediatric journals, they found that only 0.3% were RCTs ^11^, which supports our findings that a large proportion of RCTs were published in high-impact general medical journals.

One of the main limitations in this study is that it didn’t assess the quality of RCTs, so we included RCTs from our PubMed search regardless of their quality. Recent studies have stated that ophthalmology literature is of questionable methodological robustness, where RCTs become the center of the scope when methodological robustness is assessed, as they are the source of the highest level of evidence ^10, 21^. Future studies should focus on assessing quality of RCTs rather than the quantity (which was the scope of this study), where the Cochrane Eyes and Vision library criteria for RCT robustness can be utilized ^22^.

## Data availability

### Underlying data

Harvard Dataverse: Ophthalmology randomized controlled trials. https://doi.org/10.7910/DVN/TXEYDX ^23^.

This project contains the articles identified during this study.

Data are available under the terms of the Creative Commons Zero "No rights reserved" data waiver (CC0 1.0 Public domain dedication).
