# Supplementary material for: Randomized controlled trials in ophthalmology: a bibliometric study
Source: F1000Res. 2019 Oct 4;8:1718. [Version 1] doi: 10.12688/f1000research.20673.1 (PMC7156022; doi:10.12688/f1000research.20673.1)
Supplement: Peer review history

# Peer review: Reviewer response for version 1

**DOI:** 10.5256/f1000research.22737.r61423

**Published:** 2020-04-08

**Authors:** Sa'ed H Zyoud

**Affiliations:** 1 Department of Clinical and Community Pharmacy, College of Medicine and Health Sciences, An-Najah National University, Nablus, Palestinian Territory

I would like to congratulate you on the work done. This helps to analyze the most active researchers in the subject, facilitating future collaborations. I would like to ask some questions that were not very clear for me in the paper.

1. Why did you only use PubMed to survey data?
2. How did you consider collaborations?
3. You could have done more interesting things, such as a heat map (VOSViewer has a great one and is free to use - but have used it in a previous bibliometric analysis).
4. Bibliometric is an aid in the characterization of a field, but scholarly communication is too complex to explain the history, status, and development of any field of science. Bibliometric has a lot of limitations that should be recognized in order to avoid a simplistic misuse of the indicators and methodologies.
  Borgman, C. L., and Furner, J. (2002). Scholarly communication and bibliometrics. Annu. Rev. Inform. Sci. Technol. 36, 3-72. doi:10.1002/aris.1440360102 ^1^
  Hicks, D., Wouters, P., Waltman, L., Rijcke, S. D., and Rafols, I. (2015). Bibliometrics: the Leiden Manifesto for research metrics. Nature 520, 429-431. ^2^

 

  Without further comments or questions, I congratulate again the initiative.

I confirm that I have read this submission and believe that I have an appropriate level of expertise to confirm that it is of an acceptable scientific standard.

## References

[1] : Scholarly communication and bibliometrics. *Annual Review of Information Science and Technology*.2005;36(1) : 10.1002/aris.1440360102 2-72 10.1002/aris.1440360102

[2] : Bibliometrics: The Leiden Manifesto for research metrics. *Nature*.2015;520(7548) : 10.1038/520429a 429-31 10.1038/520429a 25903611

# Peer review: Reviewer response for version 1

**DOI:** 10.5256/f1000research.22737.r54690

**Published:** 2019-10-21

**Authors:** Peter Y Chang

**Affiliations:** 1 Massachusetts Eye Research & Surgery Institution (MERSI), Waltham, MA, USA

I applaud the authors' efforts in sifting through the large volume of data in determining the proportion of ophthlamology publications that are cateogorized as RCTs. The authors also found that most of the RCTs concern retinal diseases. This makes sense, as one of the most dramatic therapeutic advancements since early 2000s-  not just in Ophthalmology but all of medicine - is the employment of anti-VEGF therapy in the treatment of age-related macular degeneration and diabetic retinopathy. These two conditions, along with glaucoma, are perhaps the 3 most common ophthalmic conditions for which many newer therapeutics are being developed. It is also encouraging to see that the number of RCT published annually in Ophthalmology has generally trended upward, and that they are published in well-regarded peer-reviewed journals. Overall, though, there remains a lack of RCTs in Ophthalmology, with majority of published works being presumably retrospective in nature.

I confirm that I have read this submission and believe that I have an appropriate level of expertise to confirm that it is of an acceptable scientific standard.
